# Synergistic Effect of Paraffin-Incorporated In_2_O_3_/ZnO Multifold Smart Glazing Composite for the Self-Cleaning and Energy-Saving Built Environment

**DOI:** 10.1021/acssuschemeng.2c00260

**Published:** 2022-05-10

**Authors:** Anurag Roy, Habib Ullah, Mussad Alzahrani, Aritra Ghosh, Tapas K. Mallick, Asif Ali Tahir

**Affiliations:** † Environment and Sustainability Institute, University of Exeter, Penryn Campus, Cornwall TR10 9FE, U.K.; ‡ Mechanical and Energy Engineering Department, Imam Abdulrahman Bin Faisal University, Dammam 34212, Saudi Arabia; § College of Engineering, Mathematics and Physical Sciences, Renewable Energy, University of Exeter, Penryn, Cornwall TR10 9FE, U.K.

**Keywords:** building, composite, energy, glass, phase change, smart, thermal, wettability

## Abstract

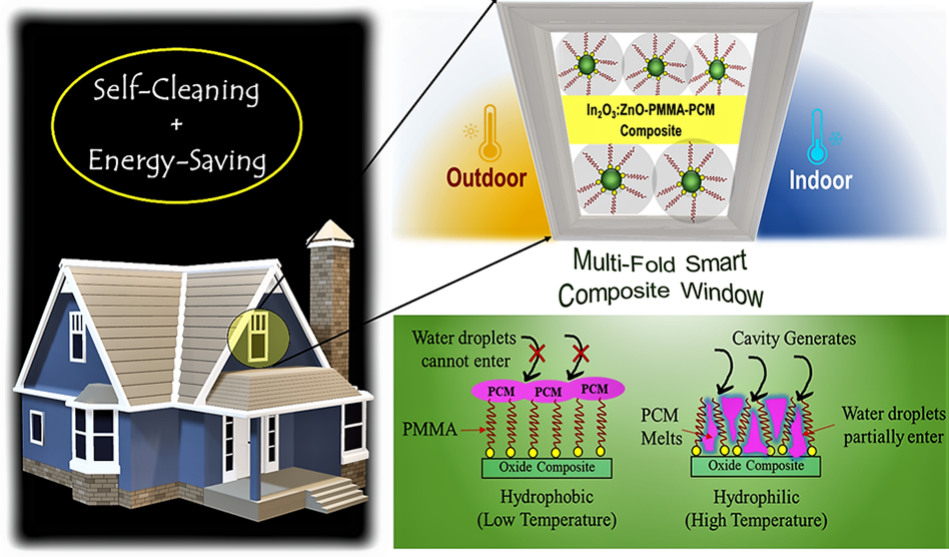

The thermal performance of window glazing requires improvement for a sustainable built environment at an acceptable cost. The current work demonstrates a multifold smart composite consisting of an optimized In_2_O_3_/ZnO–polymethyl methacrylate–paraffin composite to reduce heat exchange through the combined self-cleaning and energy-saving envelope of the smart built environment. This work has attempted to develop a smart composite coating that combines photosensitive metal oxide and phase change materials and investigate their thermal comfort performance as a glazed window. It is observed that the In_2_O_3_/ZnO (5 wt %) multifold composite film experienced better transmittance and thermal performance compared to its other wt % composite samples. Moreover, the multifold composite-coated glass integrated into a prototype glazed window was further investigated for its thermal performance, where a steady average indoor temperature of ∼30 °C was achieved when the outside temperature reached ∼55 °C, while maintaining good visibility. Interestingly, the transparency reached ∼86% at 60 °C and exhibited a hydrophobic water contact angle (WCA) of ∼138°. In contrast, a similar film exhibits ∼64% transparency at 22 °C, where the WCA becomes moderately hydrophilic (∼68°). Temperature dependency on transparency and wettability properties was examined for up to 60 cycles, resulting in excellent indoor thermal comfort. In addition, a thermal simulation study was executed for the smart multifold glazing composite. Moreover, this study offers dynamic glazing development options for energy saving in the smart built environment.

## Abstract 2

A smart window glazing composite for a sustainable energy-saving built environment enables combined indoor thermal comfort and self-cleaning features.

## Introduction

The built environment is responsible for an essential part of global energy consumption. This energy is used in thermal comfort and artificial ventilation systems.^1^ Buildings experience considerable heat loss or gain through windows, which affects the thermal comfort of buildings’ occupants.^2,3^ For a sustainable building design, windows significantly contribute to natural light consumption and building energy flows. Windows are one of the most influential building envelopes that allow external daylight and heat gain to be permitted inside the building. It also provides heat loss to flow from the building interior to the exterior depending on the external and internal indoor environments.^4^ To obtain a better indoor environment, advanced design is indispensable, maintaining a sufficient heat flow and light penetration. Traditional single or double glazing does not contribute to achieving an energy-efficient building. By varying the window size and orientation, natural light and heat flow through a window can be controlled. Multilayer, vacuum, polymer disperse, thermochromic, or phase change materials (PCMs) employed in glazing are still leading as a traditional fenestration system for indoor thermal comfort.^5−8^

Besides, windows also contribute to a building’s heating, cooling, and lighting for a better indoor environment and can be optimized through intelligent glazing, smart window glass coating design. However, their thermal performance is inferior to other building components, resulting in energy loss from the building envelope that drastically changes in the case of a large glazing area.

Various strategies are engaged in the window thermal behavior quality improvements, such as low-emission coating, thermochromic–electrochromic coating, PCM filling, and vacuum glazing.^9−11^ Spectrally selective, thermal storage, and higher transparency type parameters are critical when dealing with window glazing. However, incorporating all the desired properties in a single compound is challenging. Also, employing various combinations enlarges the glazing space and increases the cost (fabrication and maintenance) and sometimes the weight. The composite coating is the most viable option in multiple forms as a solution. PCMs are combined with oxides to produce suitable compounds for glazing, where high mechanical properties are unnecessary at the cheaper end. From the perspective of composite fabrication, incorporating metal oxides (MOs) into PCMs can exert the potential of phase change energy storage, enhancing their heat transfer performance and further improving indoor thermal comfort behavior.

On the other hand, nanostructured MOs are suitable options for their facile synthesis, high melting point, higher optical transparency, electrical conductivity, shielding effectiveness, and hard-wearing durability. They can be incorporated as an additional layer that improves the performance or enhances the glazing unit’s properties. Smart coatings can also be used within nanotechnology for the next-generation optoelectronic properties to provide ultra-lightweight, flexible, high optical transparency with infrared (IR) reflectance, and low-cost cells for extensive applications.^12,13^ Smart coatings can also generate noticeable optothermal characteristics applied to window glazing development. In_2_O_3_ is one of the leading transparent conductive oxides with high electron mobility (>62.5 cm^2^ V^–1^ s^–1^) with a high near-IR transparency.^14^ Mostly, In_2_O_3_ used as In_2_O_3_/Sn (ITO) films, prepared by magnetron sputtering, exhibits high optical transmittance and electrical conductivity.^15^ However, the fabrication process is cost-effective, and the glass’s electrical conductivity is not required for window glazing. As a result, instead of Sn doping, the high optical transparency of In_2_O_3_ offered as a suitable composite material with the PCM leads to smart composite development.

There are various photosensitive MO materials available because of their ability to modulate the throughput of light and solar energy. A high visible transmittance (83.4–85.3%) and IR reflection (up to 48.9% at 2500 nm) were achieved by Chen et al. (2015) using high-quality Ga-doped ZnO thin films due to aerosol-assisted energy efficient glazing.^16^ Sb-doped SnO_2_ glazing exhibits an average visible transmittance of 80.15% but an average near-IR (NIR) transmittance of 23.31%, promoting high NIR shielding property for energy-efficient windows.^17^ ZnO is one of the popular materials, which exhibits morphology- and microstructure-dependent optoelectronic properties through straightforward synthesis.^18^ Therefore, a precise optimization of deposition parameters within a specific methodology is required for excellent device performance.^18^

Besides, emplacement of self-cleaning glazing is also important to resist dirtying and maintain tolerable cleanliness. Both hydrophilic and hydrophobic coating options are available to create a self-cleaning property of the glass depending on their surrounding climate. TiO_2_, ZnO, SiO_2_, and ZrO_2_ are the typically available oxides.^19^ This is because switching wettability by converting the oxygen-rich state (hydrophilic) and oxygen-vacant state (hydrophobic) is possible with these oxygen-deficient oxides. Nundy et al. (2021) recently reported a ZnO-based self-cleaning coating for glazing application. Hf–ZnO films showed stable performance under real-life conditions that can be effectively utilized for windows.^20^ VO_2_-dispersed glass in a multicomponent system offers a new PCM set for excellent latent heat storage and temperature retention properties.^21^ PCM-filled double-glazed windows can reduce the entry of solar energy into buildings, while windows’ interior surface temperature can elevate up to 3.0 °C enhancement.^22^ Various experimental and simulation methods analyze the optothermal performance of a glazed unit containing PCM, including the photothermal performance of the PCM, spectral characteristics of thermal radiation, PCM scattering effect, and control of glazing system either PCM composite-coated glass or filled with the PCM composite.^23^ The first-ever optical performance of PCM-sealed glazing was investigated by Ismail and Henríquez (2002), and this was compared with an air-filled double-glazed window.^24^ It was observed that even in the translucent state, the diffuse transmission PCM-filled window showed higher visible light transmittance than the air-filled window.

Later, Li et al. (2015) also observed a similar phenomenon when glazing was filled with liquid paraffin. Using transmittance spectrograms in the 240–900 nm wavelength at normal incidence, PCM-filled windows had a higher visible light transmittance than the one filled with air.^25^ Their results showed that the paraffin material could improve the transmittance of most areas of the glazed unit. However, the PCM thickness affects the absorption and reflectance of the glazing. Furthermore, to improve the photothermal performance of a double-glazed window unit, Al_2_O_3_–TiN binary nanoparticles were successfully suspended into paraffin.^26^ Also, PCM’s enhanced photothermal properties can be achieved by adding various MO nanoparticles. PCM-graphene/graphite composite has also attracted attention as a heat transfer fluid composite. Introducing 0.5 wt % TiO_2_, ZnO, Fe_2_O_3_, and SiO_2_ can effectively enhance the thermal conductivity of PCM–MO nanocomposites by 147.5, 62.5, 55, and 45% reported by Gupta et al. (2020).^27^

However, PCM leakage is crucial for the window, which can be resolved by applying strategies like shape-stabilized composite PCMs and core–shell composite PCMs.^28^ Addition of a higher thermal conductive material can be directly introduced to the PCM to obtain higher thermal conductivity of PCMs. However, thermal conductivity enhancement of PCMs is limited and thus decelerates the thermal charging/discharging rates of PCMs. However, thermal conductivity enhancement was generally limited. To balance the optothermal properties of the glazing, PCMs can be used as a matrix with the MOs in a composite form. Traditional composite PCMs seem loose and gradually diffuse into the surface. However, the PCM is still an excellent and cheapest thermal energy storage material.

In this work, In_2_O_3_ and ZnO have been used as MO, where In_2_O_3_ contributes as a transparent MO that exhibits higher NIR transparency. In addition, ZnO facilitates wettability performance and controls the trade-off between visible-NIR optical transparency of the composite coating. Due to a high band gap (>3.5 eV), In_2_O_3_ can transmit a large portion of the visible light and is considered the leading oxide for this work.^29^ Also, instead of a different filling, paraffin (PCM) was used as a matrix to In_2_O_3_/ZnO and formed a composite. To the best of our knowledge, for the first time, we are reporting a MO–PCM multifold smart composite synthesized via a solution-processed technique. PCM incorporation within the composite can control the optothermal properties of the composite film and provide excellent temperature-dependent switching wettability characteristics. The composite glazing resulted in significant indoor thermal comfort (∼28 °C) even at 55 °C outdoor temperature. Using the composite-coated glass strategy for glazing further leads to excess energy absorption in buildings, filling the gap between energy supply and demand without using a single component and without compromising the optothermal properties of the glazing. In addition, this study creates a synergistic effect of the optothermal and wettability characteristics of the composite in terms of a smart glazing application for a sustainable built environment.

## Materials and Methods

### Materials

Indium(III) oxide 99.9997% (trace metal basis), In_2_O_3_, was purchased from Thermo Scientific. Zinc acetate, 2-methoxy ethanol, α-terpinol, and paraffin wax were supplied from Sigma-Aldrich (now Merck), U.K. Polymethyl methacrylate or PMMA (molecular weight: 15,000) was purchased from Alfa Aesar. All the chemicals were used as received without further purification.

### Preparation of IZPC Pastes

**Scheme 1:**
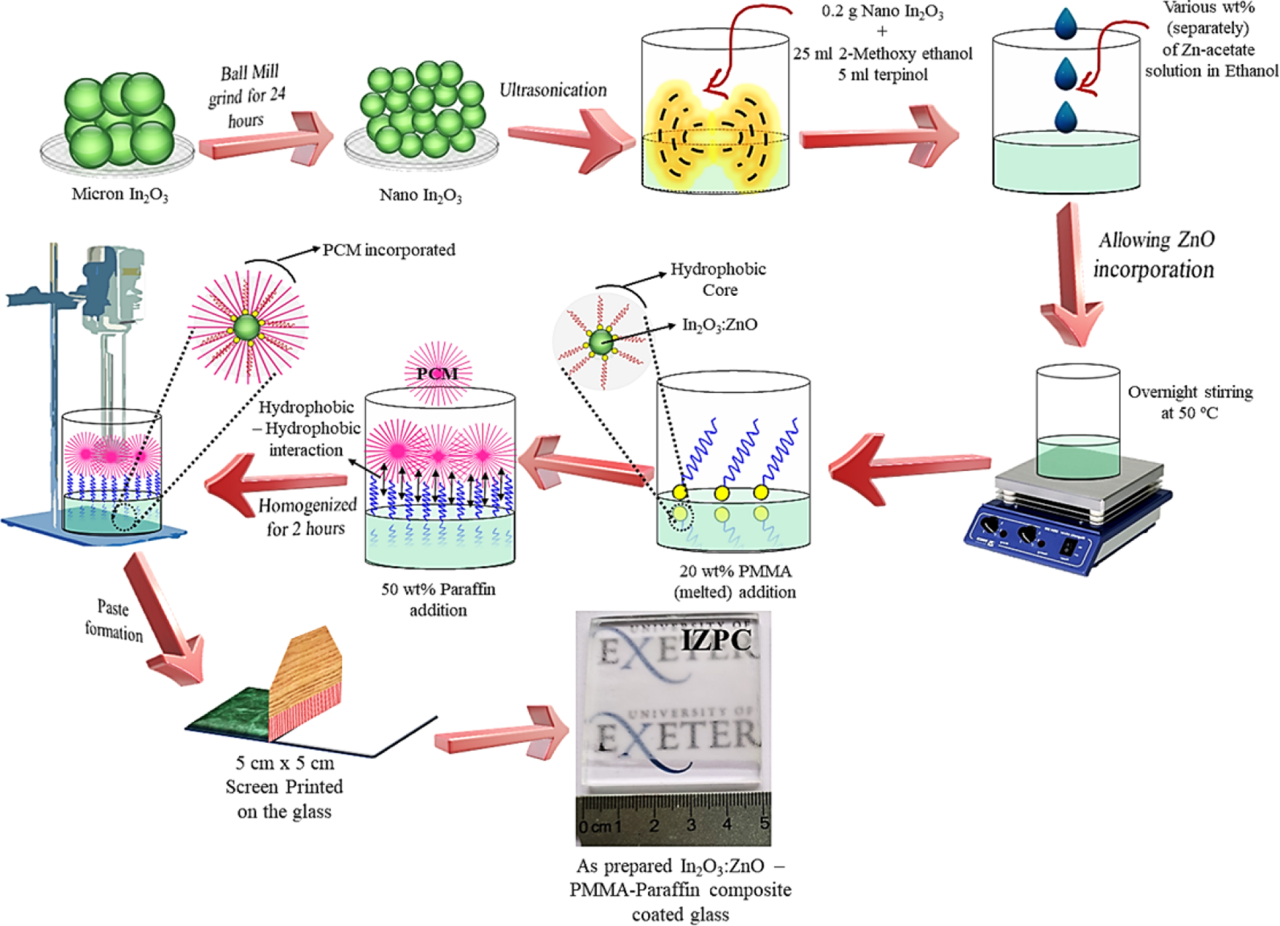
Schematic Depiction of the In_2_O_3_/ZnO–PMMA–Paraffin Multifold Composite’s Development Process (the Display Logo is Credited to the University of Exeter, U.K. and Permission is Granted for Its Display)

The solution-processed In_2_O_3_/ZnO–PMMA–PCM multifold composite synthesis and corresponding coating development are schematically represented in Scheme 1. In detail, bulk In_2_O_3_ was allowed for 24 h ball-milling to produce smaller particles (<50 nm). The collected nano-In_2_O_3_ powder (0.2 g) was mixed with 25 mL of 2-methoxy ethanol (Merck, UK) and 5 mL of α-terpinol. The solution was further ultrasonicated until it became transparent. 0.02 g of Zn-acetate was mixed with 25 mL of ethanol, leading to a colloidal solution, and added dropwise to prepare an In_2_O_3_/ZnO composite under constant overnight stirring at 50 °C. After this, 20 wt % (40 mg) of PMMA was added to the solution under continuous stirring, and the solution temperature was maintained at 60 °C. This process was undertaken to functionalize the surface of the synthesized In_2_O_3_/ZnO composite before introducing paraffin. Then, 50 wt % (0.11 g) of paraffin beads was directly added to the mixture and under a homogenizer, where the temperature was kept at 60–70 °C under constant stirring. Once the solution became translucent, the solution was transferred to a magnetic stirrer and allowed for continuous stirring for another 2 h to finalize the composite paste.

Table S1, Supporting Information demonstrates the concentration of each component in the final composite coatings and corresponding nomenclatures. The same procedure has been opted to prepare In_2_O_3_/ZnO composites at different weight percentages such as 0.05, 0.07, and 0.1 g (wt %). Therefore, the IZPC-2, IZPC-5, IZPC-7, and IZPC-10 films correspond to 2, 5, 7, and 10 wt % of Zn-doping, respectively.

### Fabrication of IZPC Coating

The composite paste was employed to fabricate its coating on the previously cleaned, UV–ozone treated (Ossila UV Ozone Cleaner) k-type glass (5 cm × 5 cm) by a screen-printing (Mascoprint, UK) method for further investigation. The coated glass was kept at 50 °C under a vacuum oven for solvent evaporation. Finally, the addition of various Zn^2+^ wt % to the In_2_O_3_ film with the paraffin composite was designated as the indium zinc oxide (In_2_O_3_/ZnO)–PMMA–paraffin composite (IZPC). The same fabrication has opted for other In_2_O_3_/ZnO composite samples.

### Material Characterization

A Bruker D8 ADVANCE X-ray diffractometer (Cu Kα irradiation, 40 kV/40 mA) was used to analyze the crystal structure of the newly developed composite films. A JEOL 2100 transmission electron microscope at 200 kV was used to obtain TEM bright-field images, high-resolution bright-field images (HRTEM), and selected area electron diffraction (SAED) of the In_2_O_3_/ZnO composite sample. A Tescan Vega3 scanning electron microscope was used to evaluate the thickness and the coating microstructure, while energy-dispersive X-ray analysis (EDX) revealed the elemental mapping of the IZPC-5 sample. A PerkinElmer LAMBDA 1050 UV/vis/NIR spectrophotometer was used to measure the optical properties of the coated and noncoated glass. Fourier-transform IR spectroscopy (FTIR) was performed using a Shimadzu IRAffinity 1S IR Spectrometer. A WITec Alpha 300R was employed to have the Raman spectrum mapping of the composite. A Shimadzu IRAffinity 1S IR Spectrometer was used for the FTIR characterization. A Bruker EMX spectrometer was used to measure the electron paramagnetic resonance (EPR) spectra at room temperature. Besides, the room-temperature photoluminescence (PL) spectra film samples were measured on the FS5 spectrofluorometer, Edinburgh Instruments.

### Thermal Performance Measurement

By employing an indoor solar simulator (Wacom AAA+, model WXS-210S-20), the thermal properties of the smart composite were evaluated under 1 sun (1000 W/m^2^) and air mass of 1.5 conditions. A Pico TC-08 (Pico Technology) data logger was used to measure the continuous temperatures.^30^ Besides, a FLIR T425 camera was used to capture the IR images of the IZPC-5 film from a distance of 10 mm. Six IZPC film samples were investigated to measure their respective transmittance spectra.

Equations 1 and 2 were employed to determine the luminous and solar transmission, respectively.^31^
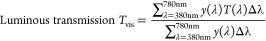
1
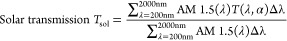
2where *T*(λ) represents the transmittance value of the composite-coated glass at wavelength λ, and *y*(λ) corresponds to human eye’s photopic luminous efficiency designated by the International Commission on Illumination (CIE). All the related transmission measurements and analyses were investigated at 520 nm compared to CIE photopic luminous human eye efficiency. Here, the human eye’s vision (*T*_vis_) limit is considered as 380 ≤ *T*_vis_ ≥ 780 nm.

## Results and Discussion

### Optical Transmission Characteristics of Various IZPC Films

**Figure 1:**
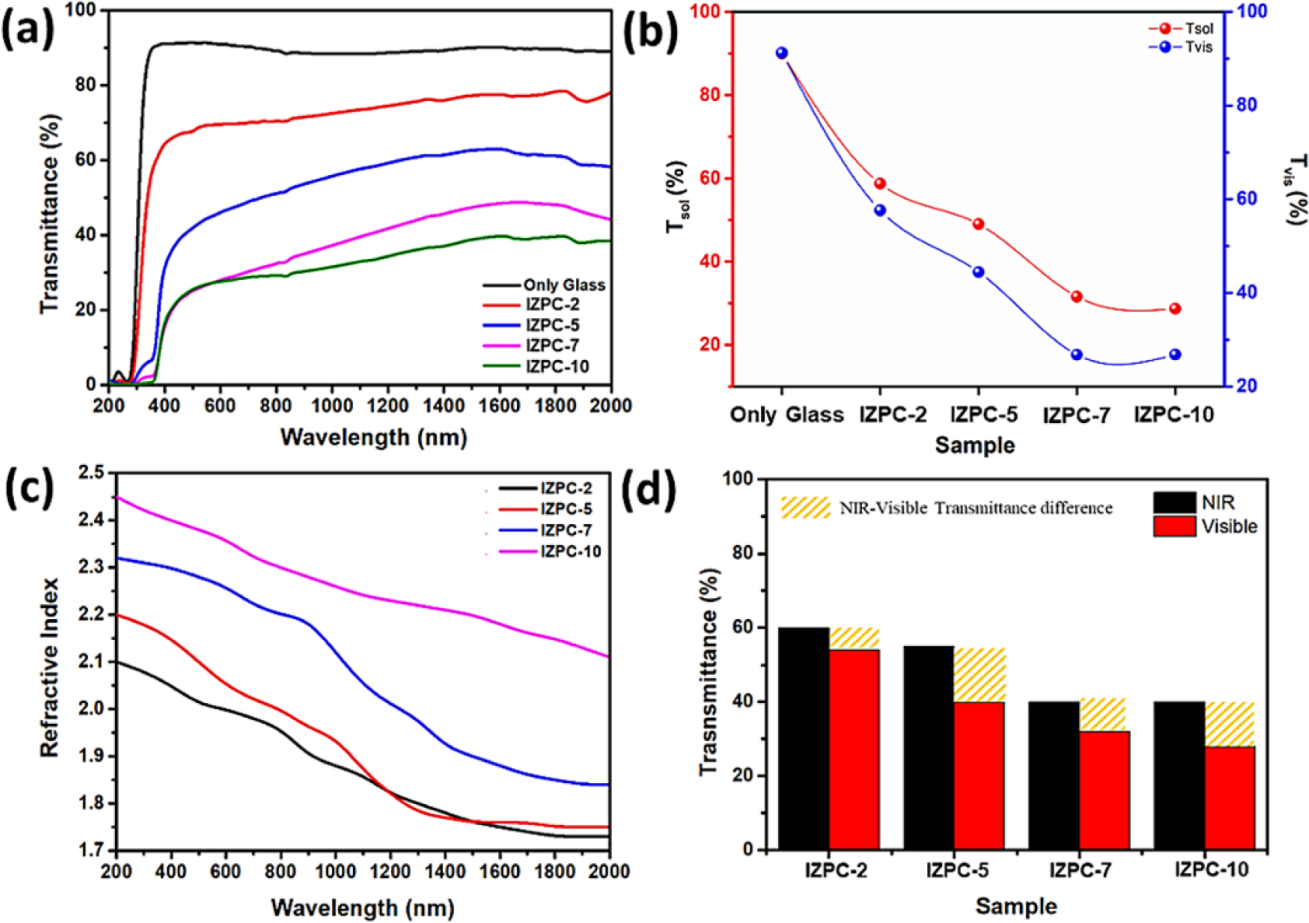
(a) Transmission spectra, (b) plot of luminous and solar transmission, (c) RI variation plot across 200–2000 nm wavelength, and (d) NIR–visible quantitative transmission difference plot for IZPC-2, 5, 7, and 10 films, respectively.

The transparency spectra of IZPC-2, 5, 7, and 10 films, along with empty glass, are shown in Figure 1a. It can be seen from Figure 1a that increasing the Zn^2+^ addition leads to lower transmission in the order of IZPC-2 > 5 > 7 >10. The visible and NIR transparency decreased with the increase of usage of Zn^2+^ due to the enhancement of coating thickness. ZnO exhibits a mild visible absorption, leading to lesser visible transmittance when increasing the wt % in the composite. ZnO addition also excels at a slight bathochromic shift ∼380 nm and further increased during wt % enhancement. As transparency plays a crucial role in solar thermal comfort, the luminous and solar transmission was measured for all the films, as shown in Figure 1b. The highest *T*_sol_ and *T*_vis_ values are recorded as quite similar as 58.74 and 57.62%, respectively, for the IZPC-2 film. The *T*_sol_ and *T*_vis_ values are proportionate with the Zn doping. The lowest *T*_sol_ and *T*_vis_ values were 28.72 and 26.82%, respectively, for the IZPC-10 film, with a difference of ∼2%. Noticeably, the difference between *T*_sol_ and *T*_vis_ values was comparatively high at ∼5% for the rest of the films. Furthermore, the refractive index (RI) was measured across the 200–2000 nm wavelength range (Figure 1c) using the following eq 3 for the composite coating.^32^
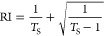
3where *T*_S_ is the percentage transmission coefficient.

The RI trend follows as IZPC-2 < IZPC-5 < IZPC-7 < IZPC -10. However, visible transparency was also lowered for IZPC-10 and IZPC-7 films, along with the NIR transparency shielding, which signifies a higher loss of overall transparency. Furthermore, to quantify the NIR and visible transparency lost for all the films, a comparative plot has been depicted in Figure 1d. The results indicate a higher difference between NIR and visible transparency, allowing for a significant shielding effect. The level of difference is in the following order IZPC-2 < IZPC-5 > IZPC-7 < IZPC-10. However, the IZPC-10 film is less transparent, as shown in Figure 1a. On the other hand, the IZPC-2 film exhibits higher transparency though the difference between NIR and visible transparency is quite low, indicating an insignificant shielding behavior. Therefore, the IZPC-5 sample was optimized that exhibits minimum trade-off between visible and NIR transparency with thickness and RI of the composite-coated glass.

### Optical Characterizations of the IZPC-5 Film

**Figure 2:**
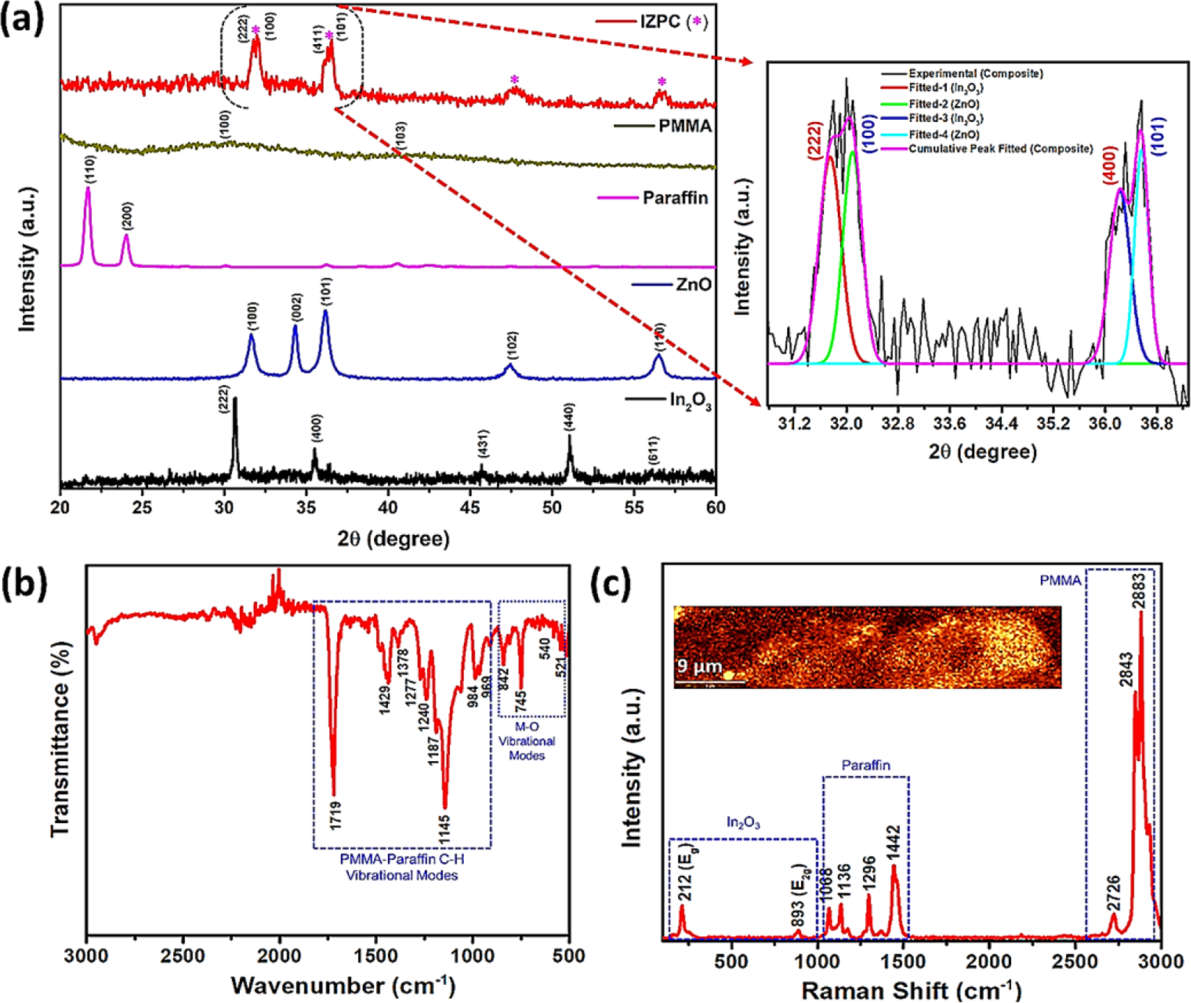
(a) XRD patterns of synthesized In_2_O_3_, ZnO, paraffin, PMMA, and their corresponding composite film samples (inset: fitted version of the composite film sample), (b) FTIR spectrum (where M = In, Zn), and (c) Raman spectrum (inset: corresponding Raman mapping image) of the IZPC-5 film, respectively.

X-ray diffraction (XRD) analysis was performed to investigate the structural metamorphosis with the IZPC-5 film, as shown in Figure 2a. The XRD pattern of the individual components of the multifold composite film was also characterized to understand the final phase composition of the synthesized composite sample.

The In_2_O_3_/ZnO–PMMA–paraffin film indicates a slight amorphous nature due to the paraffin matrix consisting of two significant intense broad peaks at 32.03 and 36.50°. The intense reflection was further fitted to understand the individual component’s contribution, where the (222) and (400) correspond to the cubic In_2_O_3_, whereas (100) and (101) planes represent the wurtzite ZnO structure, experiencing a slight shift to their original structure. To understand the phase development of the composite structure, XRD of the composite has been compared with cubic In_2_O_3_ wurtzite ZnO structures. The dominant In_2_O_3_ crystalline peak has a distance of {222} planes in polycrystalline cubic In_2_O_3_, comprehensively matching the reference diffraction patterns of JCPDS card 76-0152.^33^ At the same time, (100), (002), and (101) diffraction patterns indicate the wurtzite structure of the ZnO (JCPDS card 36-1451).^34^ This reveals that introducing the PMMA–paraffin network deteriorates the crystal phases and crystallinity of In_2_O_3_ and ZnO. However, no such promising evidence was observed for the crystal structure of the In_2_O_3_/ZnO composite in the case of IZPC-2, 7, and 10 samples. Typically, the intensities of the In_2_O_3_/ZnO composite films increase with the content of Zn^2+^ addition in the composites.

Furthermore, the FTIR spectrum of the IZPC-5 film was executed (Figure 2b), where the significant vibrational stretching modes of In–O, Zn–O, and In–O–Zn bonds were observed at the ∼500–900 cm^–1^ zone. Moreover, the intense vibrational modes correspond to ∼900–1500 cm^–1^, significantly representing the polymeric network of the PMMA–paraffin consisting of C–H bonds.^35^ No surface hydroxyl band was observed, which usually appears at >3000 cm^–1^, indicating no adsorbed water over the composite surface. Therefore, the FTIR results demonstrate successful PMMA inclusion formation and further paraffin addition into the In_2_O_3_/ZnO film, which is anticipated to create a robust chemical interaction between the π–π bonds of the C=O and the paraffin. Several chemical interactions between PMMA, paraffin, and MOs, which include π–π interactions between the M–O and PMMA skeletal and paraffin, were observed from the FTIR spectrum. The intense Raman active band at ∼212 and ∼893 cm^–1^ of E_g_ symmetry corresponds to the typical cubic In_2_O_3_ structure originated due to the symmetric stretching of the In–O bonds shown in Figure 2c. The corresponding Raman mapping inset of Figure 2c also highlights the homogeneous distribution of the composite. The other phonon modes of the IZPC-5 film appeared at ∼1068, 1136, 1296, and 1442, assigning for the C–C skeletal stretch, C–C stretch, CH_2_ deformation, and CH_2_ deformation of the paraffin structure. The Raman band appearing at ∼2726 represents the combination band involving O–CH_3_ mode; at ∼2843 and ∼2883 cm^–1^, signals confirmed the presence of the (C–H) of O–CH_3_ with (C–H) of α-CH_3_ and (CH_2_) modes of the PMMA structure.^36^

### Transmission Electron Microscopic Study of 5 wt % In_2_O_3_/ZnO Sample

**Figure 3:**
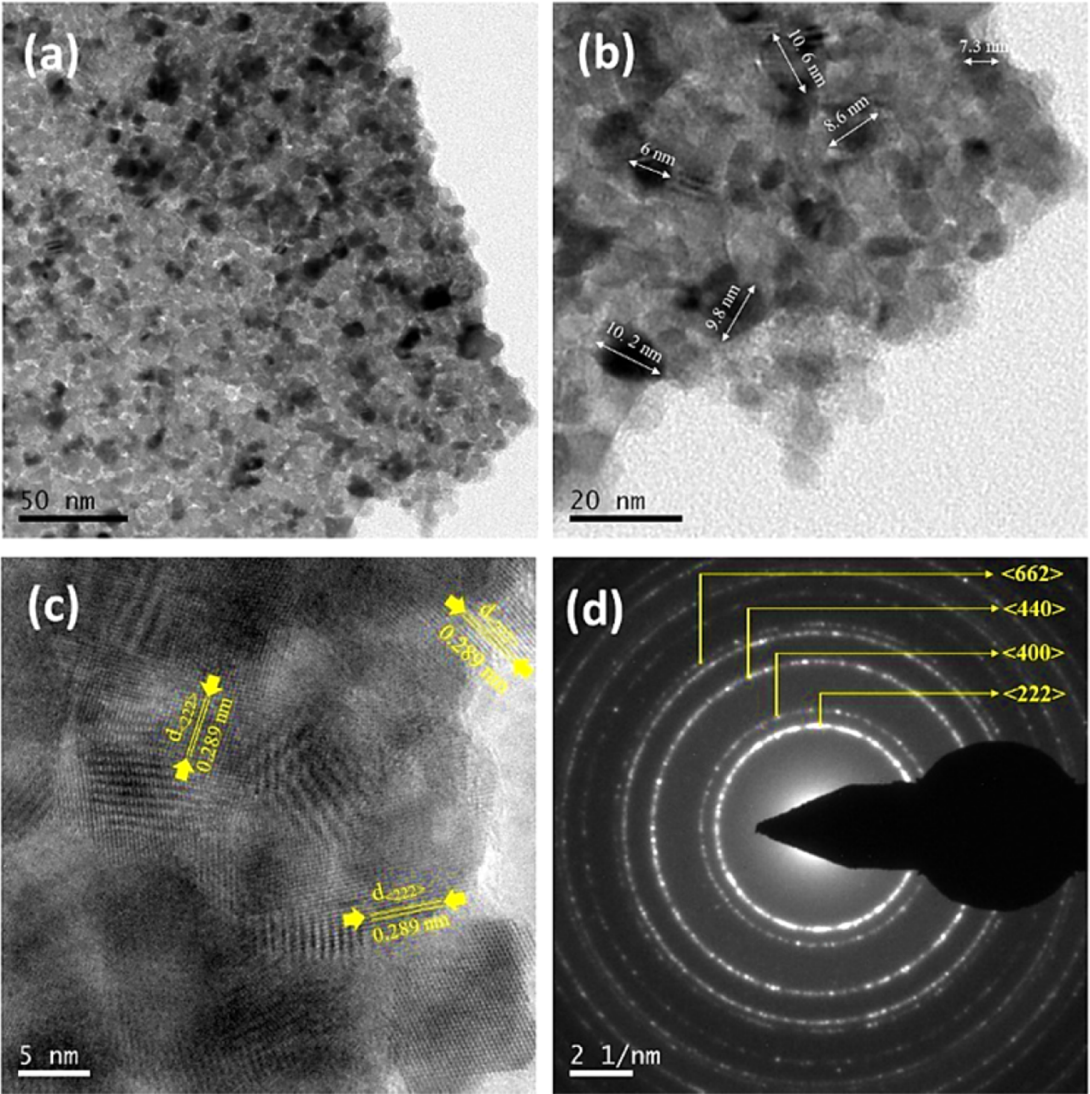
(a,b) TEM bright images of 5 wt % ZnO/In_2_O_3_ at different magnifications, (c) corresponding HRTEM, and (d) SAED pattern images.

The TEM images reveal that the ball-milled synthesized In_2_O_3_ has an agglomerated nanoparticle shape, as shown in Figure 3a,b. The distributed average particle size is ∼8 nm. The corresponding HRTEM images (Figure 3c) indicate the *d* spacing of ∼2.89 Å, which signifies the (222) crystal plane of the cubic In_2_O_3_ lattice. The SAED pattern revealed a distinct cubic In_2_O_3_ lattice, which indicates the highly crystalline nature of the nanoparticles, as shown in Figure 3d.

Interestingly, neither the HRTEM nor the SAED pattern signifies the appearance of wurtzite ZnO planes. The results reveal that Zn^2+^ that has been added within the In_2_O_3_ structure may result in amorphous ZnO formation. After comparison with the XRD, Raman, and TEM analyses, it is challenging to claim ZnO incorporation in the In_2_O_3_ structure. However, Figure 2a highlights a weak (101) plane that leads to amorphous wurtzite ZnO. Furthermore, to confirm the presence of ZnO, the EPR analysis of the composite powder was employed, signifying the intrinsic defect center present in the composite compared to the In_2_O_3_ sample. Figure S2a, Supporting Information indicates that a characteristic EPR line at *g* = 1.954 for the composite compared to In_2_O_3_, which may originate from oxygen or zinc vacancies or interstitials present in ZnO, strongly signifies the presence of ZnO in In_2_O_3_ instead of Zn^2+^ doping.

To further confirm this, a 5 wt % In_2_O_3_/ZnO sample was considered for PL analysis. Figure S2b, Supporting Information exhibits an intense near-band edge emission ∼385 nm along with a broad visible PL band ∼582 nm, indicating characteristic features of ZnO present in the In_2_O_3_/ZnO structure compared to only In_2_O_3_ when excited at 350 nm.

### Scanning Electron Microscopic Study of IZPC-5 Film

**Figure 4:**
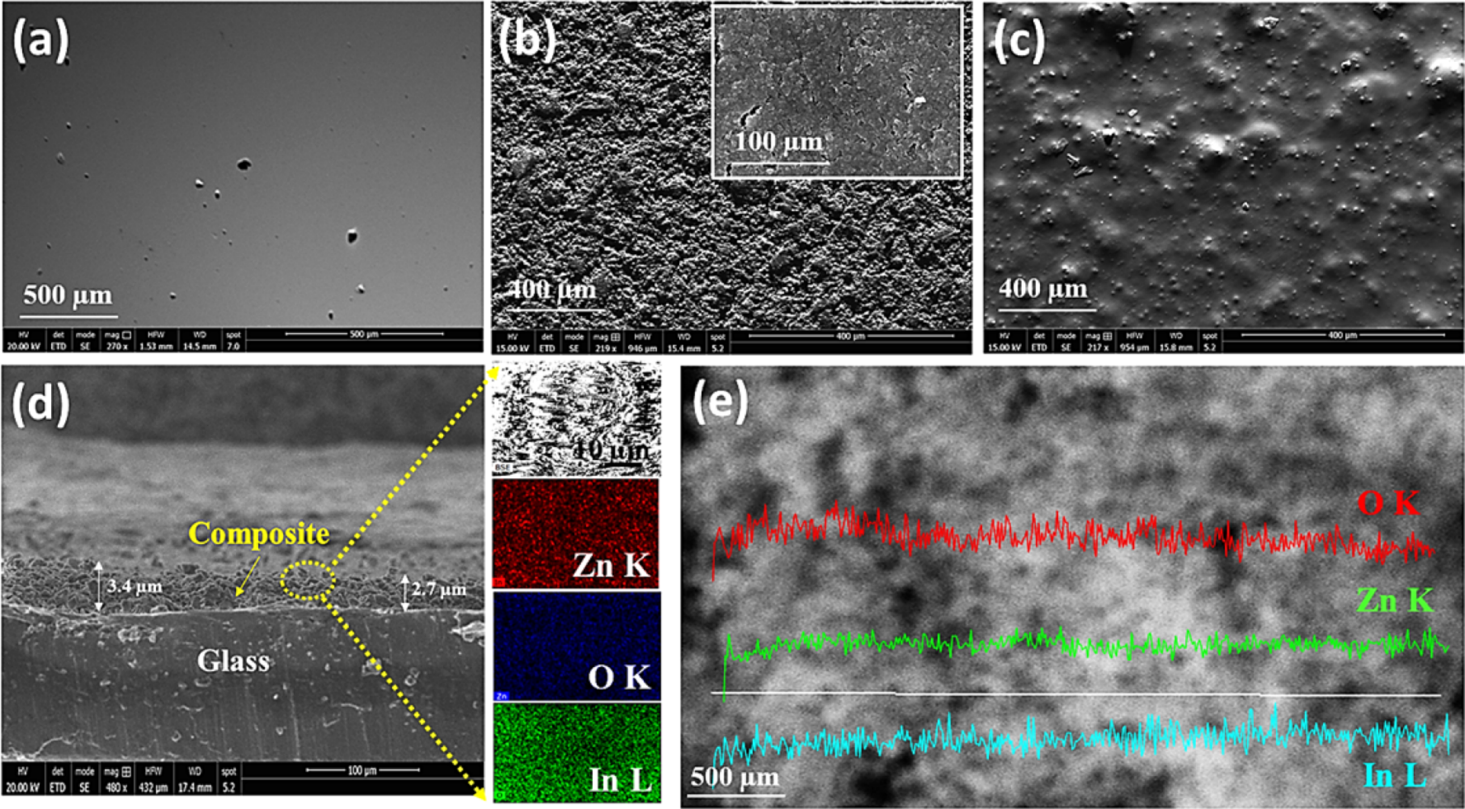
SEM microstructural images of (a) empty glass (without composite coating), (b) In_2_O_3_/ZnO (5 wt %)–PMMA film, (c) In_2_O_3_/ZnO (5 wt %)–PMMA–paraffin film, respectively, (d) SEM cross-sectional image and EDX elemental profile of the In_2_O_3_/ZnO–PMMA–paraffin film, and (e) corresponding EDX elemental distribution line-mapping across the thickness.

The SEM images of composite films are shown in Figure 4a–e. Figure 4a represents the SEM image of the empty glass. Figure 4b exhibits the 5 wt % ZnO/In_2_O_3_ composite with well-distributed spherical-type particles once screen-printed on the glass. Figure 4c highlights a thick chunk pattern of the PMMA- and paraffin-incorporated oxide composite structure, creating a few grain boundaries.

However, due to paraffin incorporation, the composite does not provide particular morphology or orientation. The average thickness of the coating is measured as ∼3.05 μm, as observed from the cross-sectional SEM image of the IZPC-5 film (Figure 4d). Besides, a homogeneous distribution of In, Zn, and O, as leading elements, was observed across the coating thickness evaluated up to 500 μm, as shown in Figure 4e, indicating successful deposition of the composite coating on the glass.

### Opto-Thermal Cycle Test of the IZPC-5 Film

**Figure 5:**
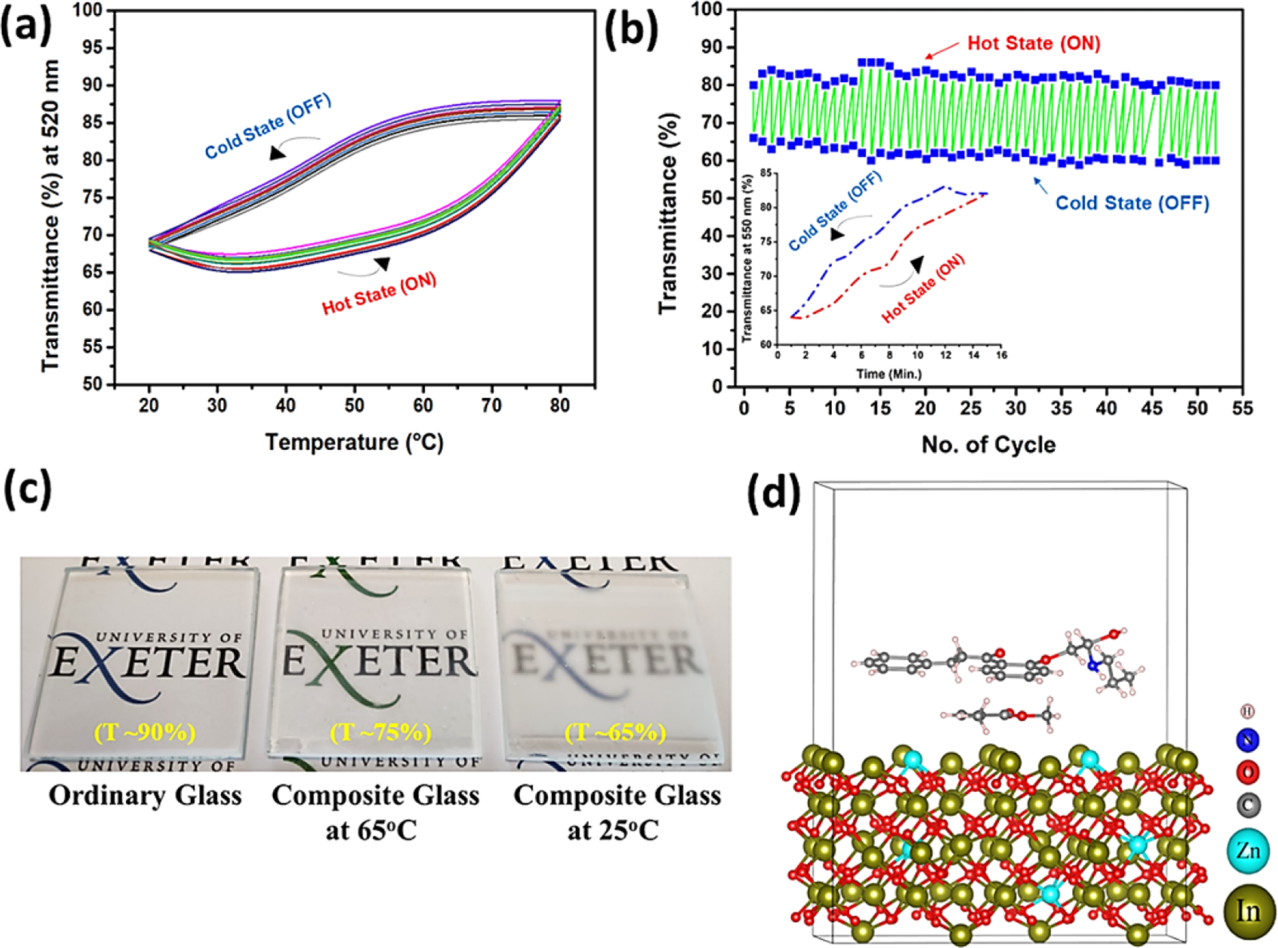
(a) Temperature vs transmission cyclic stability plot, (b) corresponding cycle plot (inset: time-dependent optical transmission plot for one cycle), (c) photograph indicating temperature and transmission state of the IZPC-5 film compared to ordinary glass (T stands for transmission), respectively, and (d) schematic representation of the In_2_O_3_/ZnO (5 wt %)–PMMA–paraffin composite structure (the display logo is credited to the University of Exeter, U.K. and permission are granted for its display).

The IZPC-5 film is further considered for thermal treatment to observe its transparency across various temperatures. Figure 5a represents the cycle plot originating from the temperature and transparency variation for the IZPC-5 film. The temperature was enhanced from 22 to 80 °C, allowing for a steady transparency enhancement of the film from its translucent state, where ∼68% transmission was recorded, to the transparent state, signifying ∼88% of transmission. On the other hand, during the temperature release from 80 to 22 °C, the same film experienced a steady decrement in its transparency from ∼88 to ∼68%, that is, almost the reverse case of temperature enhancement. The overall experiment forms a highly repeatable cycle. The comprehensive testing was performed for up to 50 cycles, reflecting the temperature on the composite film’s transparency tuning, as shown in Figure 5b. The film’s optical transparency study is also shown in Figure 5b, which signifies one cycle covering the transparent (∼85%) to translucent (∼65%) state and vice versa of the composite film taking ∼30 min.

The corresponding photograph (Figure 5c) of the composite film exhibits temperature depending on the transmission states of the composite film. The incorporation of paraffin changes the transparency due to its phase-changing thermal storage property. However, the novel observation of this study lies in paraffin inclusion with the oxides never becoming opaque on temperature treatment that avoids complete transparency loss, mostly occurring with the PCM. The theoretical model of the composite is illustrated in Figure 5d. From Figure 5d, it can be inferred that In_2_O_3_/ZnO (5 wt %) might have a strong electrostatic interaction with PMMA and paraffin. Furthermore, the hydrophobic core of the PMMA triggers paraffin hydrophobic–hydrophobic interaction and finally produces the composite. The addition of PMMA here is critical because of paraffin addition, and at the same time, PMMA protects the composite from paraffin leakages during its phase transition.

### Temperature-Dependent Switchable Aqueous Wettability Study of the IZPC-5 Film

**Figure 6:**
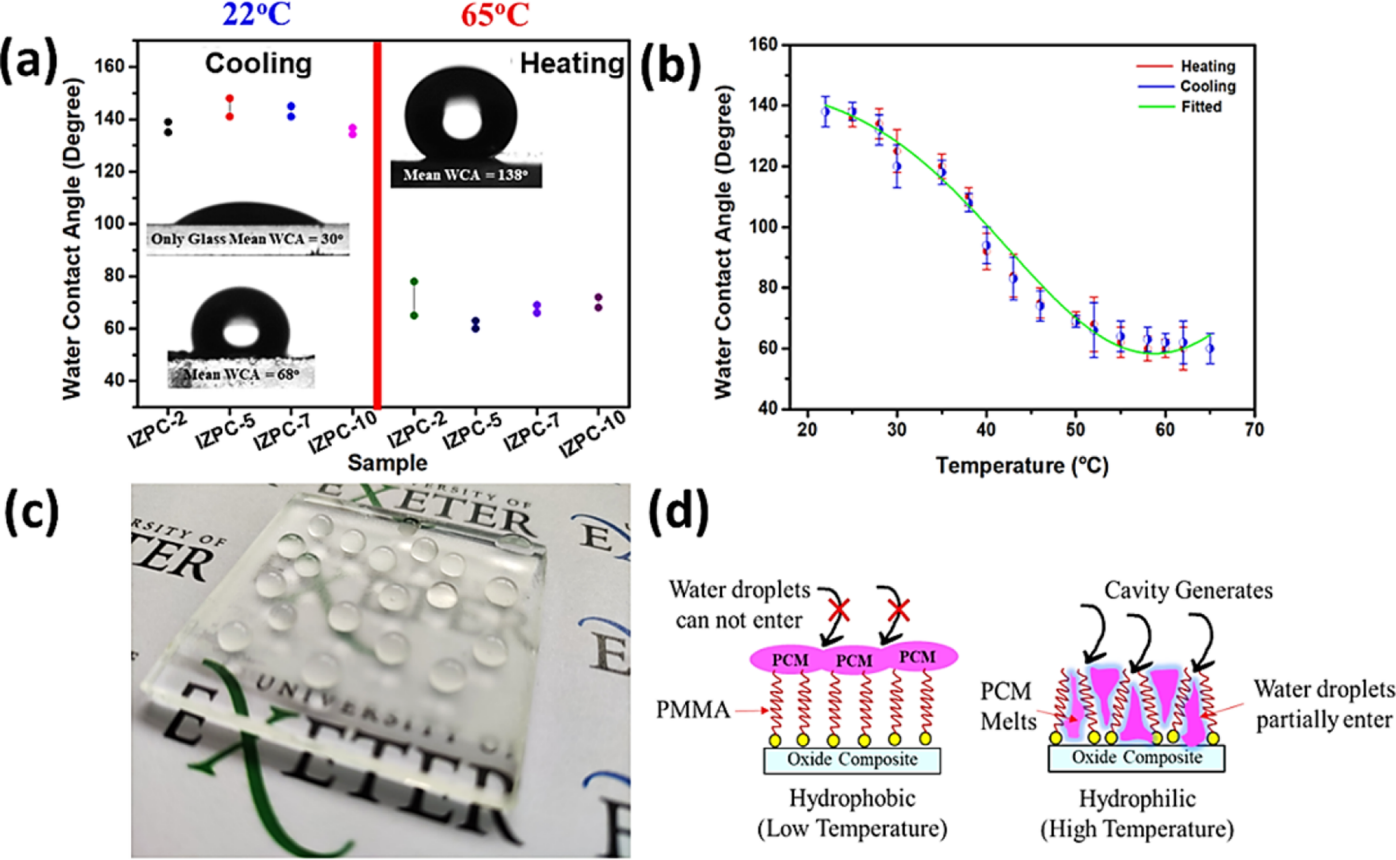
(a) WCA measurement plot for various IZPC films corresponding to their respective heating and cooling states, (b) temperature-dependent switchable WCA plot for the IZPC film, (c) photograph of the IZPC-5 film, representing the hydrophobic behavior of the coated film (the display logo is credited to the University of Exeter, U.K. and permission are granted for its display), and (d) schematic representation of the switchable wettability characteristics of the IZPC composite.

Besides the transparency tuned property, the IZPC films have switchable wettability behavior that can be projected for self-cleaning coating purposes. Figure 6a exhibits the water contact angle (WCA) measurements for all the IZPC films in their heating and cooling states. The composite film shows remarkable switchable wetting characteristics depending on the temperature treatment and transparency. Interestingly, the IZPC-5 film exhibits the maximum WCA of ∼138° once treated at 65 °C. The corresponding WCA photographs have been included in Figure 6a, where the WCA variation of the composite film can be seen concerning the empty glass.

In contrast, the WCA reduced to 68° at 22 °C. Also, among the WCA difference between the heating and cooling states of all IZPC films, IZPC-5 exhibits the highest WCA difference. The switching WCA characteristics of the IZPC-5 film follow a sinusoidal WCA variation concerning different temperatures, as shown in Figure 6b. During the heating and cooling period, the IZPC-5 film illustrates almost negligible changes in its WCA and can be tuned across the temperature multiple times. A photograph of the IZPC-5 films is shown in Figure 6c, where the hydrophobic behavior of the coating surface is visible. Primarily, paraffin is hydrophobic, and as a result, the coating becomes hydrophobic at room temperature.

The hydrophilic surface of the composite coating permits more natural light to pass through and diminish any pollutants through photocatalytic reaction and antisoiling properties. In contrast, the hydrophobic surface of the composite layer can reduce corrosion, ice, and drag reduction. On the other hand, at a higher temperature, paraffin transforms to its melting state, which facilitates cavity formation, allowing for water ingression and thus resulting in a moderate hydrophilic nature.^37,38^ A schematic representation of the switchable wettability phenomena of the composite is represented in Figure 6d. The composite-coated glass can be used as a bow window, casement window, awning window, roof-garden windows, and hopper windows, depending on the climate of the composite-coated glass.

### Prototype Glazing Testing of the IZPC-5 Film for Indoor Thermal Comfort

**Figure 7:**
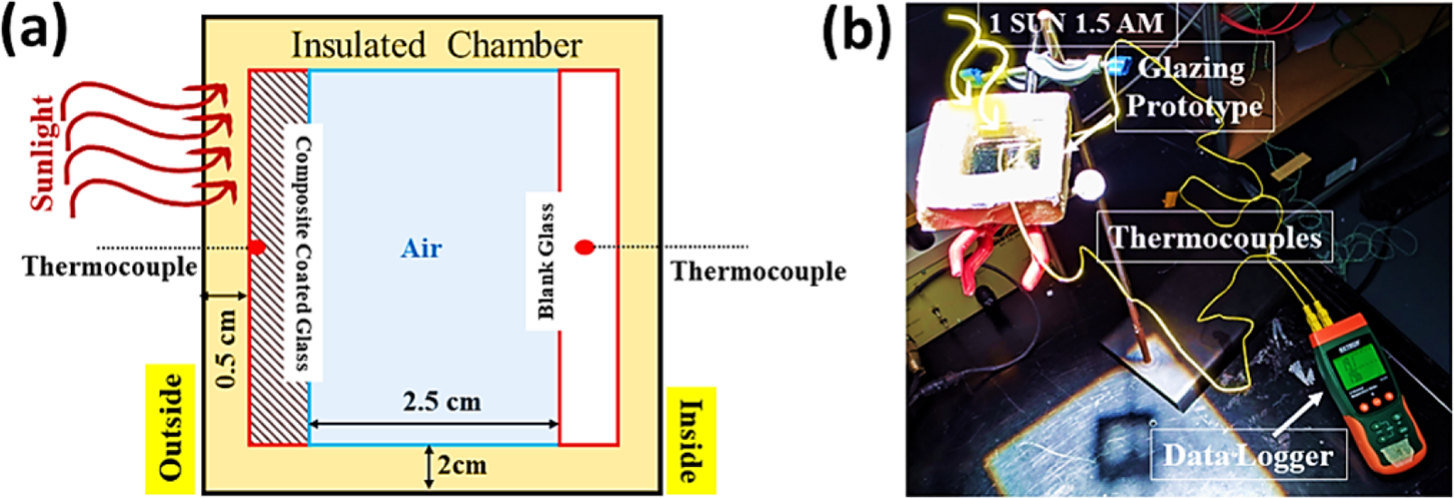
(a) Schematic representation of the IZPC-coated glass’s temperature profile measurement through a prototype window glazing setup. Glazing setup for temperature measurements and (b) photograph of the experimental setup, where the IZPC-coated glass is placed under 1 sun and connected with a data logger with thermocouples for its temperature profile measurements.

The composite films were further investigated for their temperature measurements under 1 sun 1.5 AM in the prototype window. Figure 7a represents a schematic representation of the overall testing process for the composite coated glass. I. The surface temperatures of the top and bottom glasses were measured, which are kept at a distance of 2.5 cm, filled with the air, and sealed by a 5 cm thick insulation material (Celotex GA4000) throughout the experiment to understand their temperature profile.^39^ The coated side of the glass is in contact with the outside of the glazing confined in a carefully sealed double-glazed window. The recorded temperature of the composite-coated glass was measured, heading it toward the light source, and considered outdoor.

Furthermore, Figure 7b represents the experimental setup photograph for thermal performance analysis of the composite coating on the glass. Experiments were performed for 200 min, including 60 min of continuous 1 sun exposure and 140 min without light exposure. In the presence of light, the coating temperature rises, attaining ∼86% transparency, while it becomes translucent (∼68% of transmittance) at a room temperature of 22 °C.

**Figure 8:**
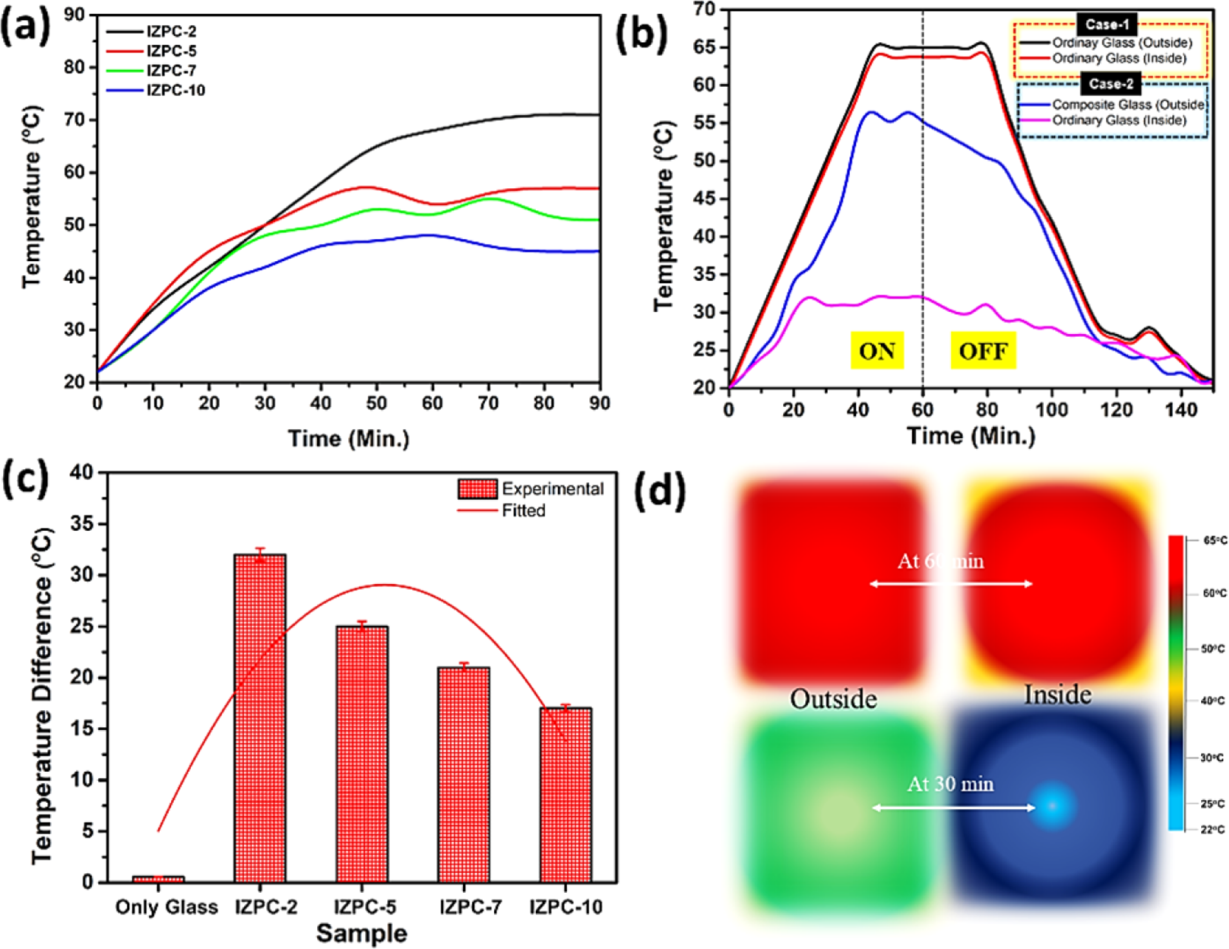
(a) Time-dependent surface temperature profile for IZPC composite films, (b) temperature profile of the IZPC-5 film compared with the ordinary glass, (c) temperature difference plot of various composites, and (d) time expended on IR thermal processed images of the IZPC-5 film compared with the ordinary glass.

The time-dependent surface temperature profile for all the IZPC films is shown in Figure 8a. On increasing the temperature, the paraffin melts, resulting in transparency enhancement for all the cases. During the emplacement of composite glass on the outside, the trade-off between transparency and temperature is crucial for thermal comfort on the inside. The time-dependent surface temperature characteristics of all the IZPC films follow a steady temperature enhancement of up to ∼40 min. Interestingly, the temperature does not rise significantly for IZPC-5, 7, and 10 films, whereas a continuous steady temperature enhancement was observed for the IZPC-2 film. A higher number of In_2_O_3_/ZnO–paraffin films restrict the higher temperature enhancement; however, the transparency is also lowered. Comparing the temperature enhancement restriction and higher order of transparency, the IZPC-5 films were well fitted for the different glazing characteristics.

Figure 8b illustrates the IZPC-5 film’s thermal profile when facing the external lab environment (directly facing the 1 sun exposure). After 40 min of continuous exposure, the composite-coated glass surface temperature reached ∼55 °C. During this process, the internal ambient temperature was always maintained at an average temperature of ∼28 °C, demonstrating thermal comfort (case-2). Under no light exposure conditions, the composite glass temperature continuously dropped, and after 140 min, it achieved thermal equilibrium when the internal and external surfaces had no temperature difference. A similar experiment was performed on the ordinary glass with no composite coating. For this regular glass, the internal ambient temperature was insufficient to attain comfort, as shown in Figure 8b (case-1).

The IZPC-2 sample demonstrates the maximum temperature difference across the glass panes due to its high outdoor surface temperature consumption. The temperature difference between external and internal surfaces was recorded for 200 min for both composite coated and noncoated glass, as shown in Figure 8c. These results signify that the composite-coated glass has enhanced thermal properties, making it suitable for building window applications and enhancing thermal comfort.

In comparison, IZPC-5 exhibits an average temperature difference of ∼25 °C. Increasing the Zn^2+^ wt % reduces the transparency of the film, thereby restricting the incoming temperature enhancement and resulting in lesser temperature differences than observed for the IZPC-7 and 10 films. Figure 8d represents the IR thermal processed images of the IZPC-5 sample compared with the ordinary glass. In the case of regular glass, the negligible temperature difference was observed even at 60 min, whereas employing a composite glass provides excellent indoor thermal comfort even at 30 min of exposure. In contrast, the air between the glazing panes requires internal cleaning as dust in the air can create scattering and reduce the visibility through the glazing, effectively reducing the daylight control and sometimes becoming insufficient for prolonged use.

PMMA addition is significant to designing and developing a PCM-incorporated composite. This is because paraffin leakage can be prevented if there is a cover of PMMA on it, and at the same time, PMMA provides a strong interaction with the PCM through their hydrophobic interaction. During the phase transition, paraffin within the composite did not suffer any kind of leakage due to the presence of PMMA. To confirm this, theory composite weight was measured throughout the 7-day period, as shown in Figure S3a, Supporting Information. After precise measurement of the composite film in its transparent state, no weight loss was observed, indicating no leakage of paraffin (Figure S3b, Supporting Information). Hence, it can be established that this work has successfully developed a smart composite film that is multifunctional and holds no leakage from the paraffin during its phase transition and offers highly energy-efficient properties for smart window applications.

### COMSOL Simulation Study of the IZPC-5 Glazing

The simulation followed the temperature measurements for the ordinary glass and IZPC-5 film to validate the conducted measurement approach. The COMSOL Multiphysics numerical tool simulated the performance based on the ray optics and heat transfer in solid models.^40^ The solution relied on a bi-directionally coupled ray-tracing study. This study solves for the ray optics model at conditions similar to the one under a Wacom AAA+ continuous solar simulator (model WXS-210S-20) (1000 W/m^2^—1.5 AM). Then, the results of the ray optics model in terms of deposited heat power are used to solve for the heat source (*Q*_s_) terms in the heat transfer balance equation in the heat transfer model, as in eq 4
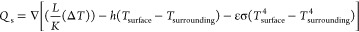
4where (∇) is the delta operator for (*x*, *y*, *z*), *L* is the layer thickness (m), *K* is the thermal conductivity W/m·K, *h* is the convective heat transfer coefficient (W/m^2^·K), ε is the thermal emissivity, and σ is Stefan–Boltzmann constant (5.67*E* – 8 W/m^2^·K^4^).

**Figure 9:**
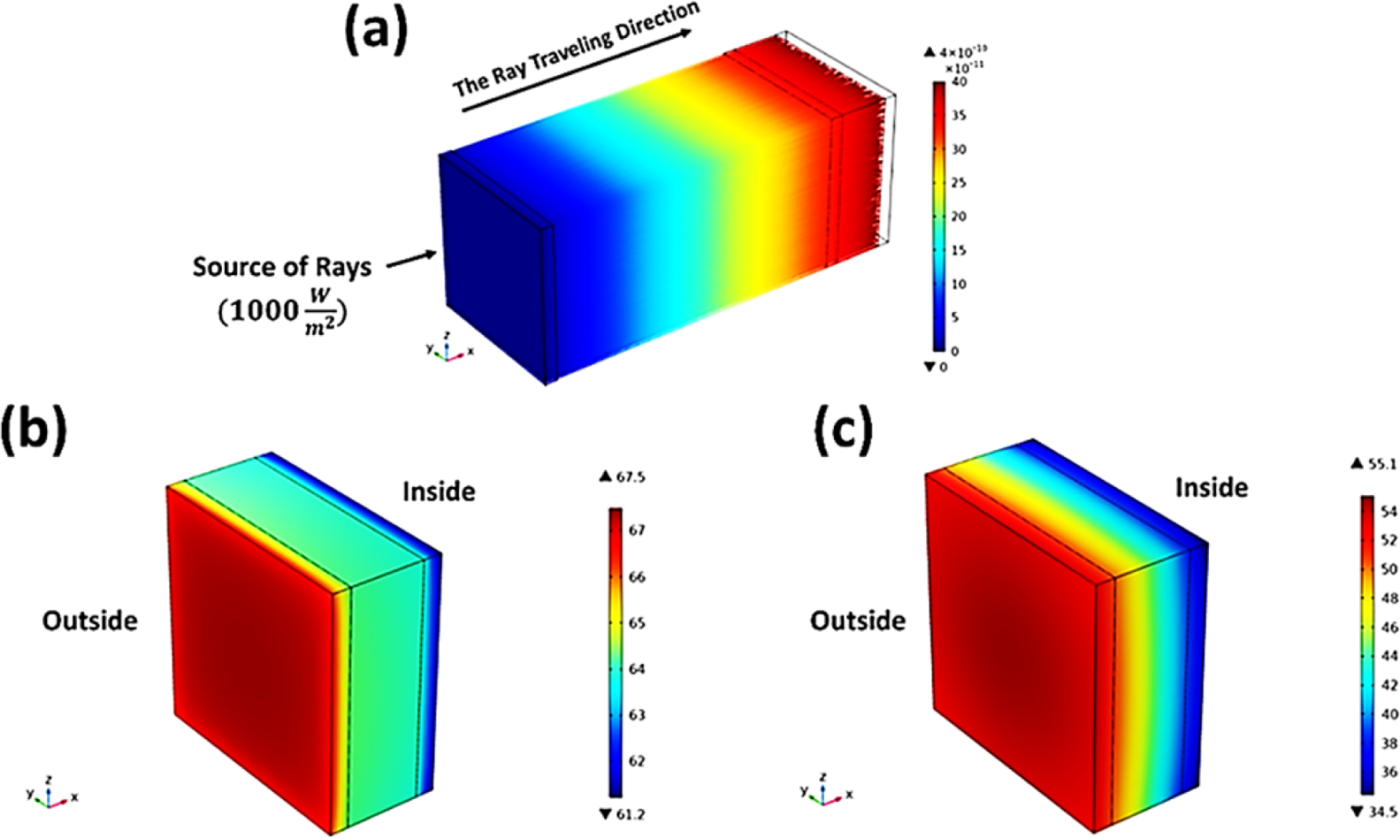
COMSOL-Multiphysics simulation results where (a) ray trajectory in nanosecond (ns), (b) temperature stratification results for the ordinary glass in °C, and (c) temperature stratification results for the IZPC-5 film in °C.

Figure 9a shows the simulated three-dimensional (3D) ray trajectories and temperature profile for the prototype glazing. The discrepancy in temperature for the ordinary glass between measurements and simulation results is found to be ∼65 and ∼67 °C for outside and inside the glass, respectively (Figure 9b). On the other hand, the discrepancy in indoor temperature for the IZPC-5 film between measurements and simulation results is observed at ∼28 and ∼34 °C (Figure 9c).

In_2_O_3_ is an n-type semiconductor, a moderate wide band gap (3.0–3.5 eV) semiconductor with high transparency in the visible and near IR region having high electrical conductivity.^41^ A heterogeneous structure is possible by incorporating MO semiconductors with other MOs, which leads to type II heterojunctions having photocatalytic behavior.^42^ Containing a higher number of oxidative radicals, both ZnO and In_2_O_3_ create charge carriers at their interface may facilitate their photocatalytic performance. Besides, at a higher temperature, due to the increased transparency of the composite coating, light waves pass through and diminish any pollutants through photocatalytic reaction and antisoiling properties.

This behavior may manifest a combination of photocatalytic and self-cleaning coating that can reduce the cleaning cycles to create savings in personnel costs. This synergistic composite coating can be recommended for photovoltaic (PV) glazing as a simplified, cost-effective solution for the dust element. The dust element has a substantial impact on reducing PV power and efficiency and can restrict unwanted thermal stress of the PV unit.^43^ Reduction of light transmission because of dirt and grime is possible to eliminate for glazing and translucent membrane application.

## Conclusions

In a building, windows are majorly responsible for most of the building energy flows and thus recommend sustainable building design to evolve an energy-saving built environment. This work has implemented an effort to explain multifold composite development that includes various window glass components, ensuring energy savings. The In_2_O_3_/ZnO–PMMA–paraffin composite was synthesized by employing a solution-processed low-cost technique to develop a novel smart window. This will have PCM property that will tune the transmission, control the solar heat penetration, and possess a transparent IR absorber on the glass. Incorporating the paraffin PCM with In_2_O_3_/ZnO offers a unique coating approach that reduces the necessity of filling between the glass panes. The XRD, IR spectroscopy, and Raman characteristics of the composite film further stipulate the successful formation of the composite. The trade-off between transparency and high temperature retarding was scrutinized for the 2, 5, 7, and 10 wt % of In_2_O_3_/ZnO composite films, where the 5 wt % film was the most efficient. The scanning electron microscopy study of the composite film further confirmed the homogeneous nature of the coating, resulting in an average 3 μm thickness. The composite coating experienced temperature depending on transparency, where the highest transparency was recorded as 88% at 65 °C, whereas the lowest transparency reached 68 °C when the temperature was 22 °C. The transparent–translucent characteristics of the coating are highly repeatable, as observed in up to 50 cycles. Besides the transparency tuning, the composite exhibits excellent switchable wettability features that show lower temperatures’ hydrophobic characteristics. The encapsulation of PCMs with PMMA is an effective strategy to improve the thermophysical properties and prevent PCM leakage.

The composite glass was further employed for the glazing analysis using its temperature profile observation, and the ray optics simulation was performed using the COMSOL Multiphysics numerical simulation model. The experimental and simulation results corroborate the multifold composite glass outdoor observations, and the indoor temperature exhibits excellent thermal comfort. In contrast, the wettability shifted to moderate hydrophilic behavior at higher temperatures. Also, the transmission tuning and switching wettability characteristics of the In_2_O_3_/ZnO–PMMA–paraffin composite revealed a synergistic effect that can be further successfully applied for the self-cleaning and energy-saving smart built environment.

However, a few critical parameters such as coating fabrication techniques, composite thickness and viscosity, coating homogeneity, and lower temperature performance must be optimized, ensuring the best natural lighting conditions with no glare. Moreover, this work is to develop technology based on state-of-the-art techniques to enable end users to reduce energy consumption, while balancing visual comfort with hygrometric wellbeing.

## Supporting Information Available

Concentration of each component in the final composite coatings and corresponding nomenclatures; EPR and PL spectra of the In_2_O_3_/ZnO (5 wt %) sample; and daywise weight loss investigation for the IZPC-5 sample along with their photographs (PDF)
