# Supplementary material for: Synergistic Effect of Paraffin-Incorporated In2O3/ZnO Multifold Smart Glazing Composite for the Self-Cleaning and Energy-Saving Built Environment
Source: ACS Sustain Chem Eng. 2022 May 10;10(20):6609–21. doi: 10.1021/acssuschemeng.2c00260 (PMC9131515; doi:10.1021/acssuschemeng.2c00260)
Supplement: Supplementary file 1 — sc2c00260_si_001.pdf [file sc2c00260_si_001.pdf]

Supporting Information Only

Synergistic Effect of Paraffin Incorporated In_2O_3:ZnO Multi-Fold Composite Smart Glazing for the Self-Cleaning and Energy-Saving Built Environment

Anurag Roy,[1,*] Habib Ullah,[1] Mussad Alzahrani,[1,2] Aritra Ghosh,[3] Tapas K. Mallick[1] and Asif Ali Tahir[1,*]

[1]Environment and Sustainability Institute, University of Exeter, Penryn Campus, Cornwall TR10 9FE, U.K.

[2]Mechanical and Energy Engineering Department, Imam Abdulrahman Bin Faisal University, Dammam 34212, Saudi Arabia.

[3]College of Engineering, Mathematics and Physical Sciences, Renewable Energy, University of Exeter, Penryn, Cornwall, TR10 9FE, U.K.

*Corresponding Authors:

A.Roy30@exeter.ac.uk; ar.chem30@gmail.com (A.R.)

A.Tahir@exeter.ac.uk (A.T.)

There is a total of 3 (three) pages, 3 (three) figures and 1 (one) table in the supporting information.

**Table S1.** The concentration of each component in the final composite coatings and corresponding nomenclatures.				
Sample Name	Amount of In_2O_3 in 25 ml (g)	Amount of colloidal Zn-acetate solution in 25 ml (mg)	Amount of PMMA# (mg) in 25 ml	Amount of paraffin beads* (g) in 25 ml
IZPC-2	0.2	20	44	0.110
IZPC-5	0.2	50	50	0.125
IZPC-7	0.2	70	54	0.135
IZPC-10	0.2	100	60	0.150
#20 wt% of In_2O_3:ZnO for PMMA<br>*50 wt% of In_2O_3:ZnO for Paraffin (PCM)				

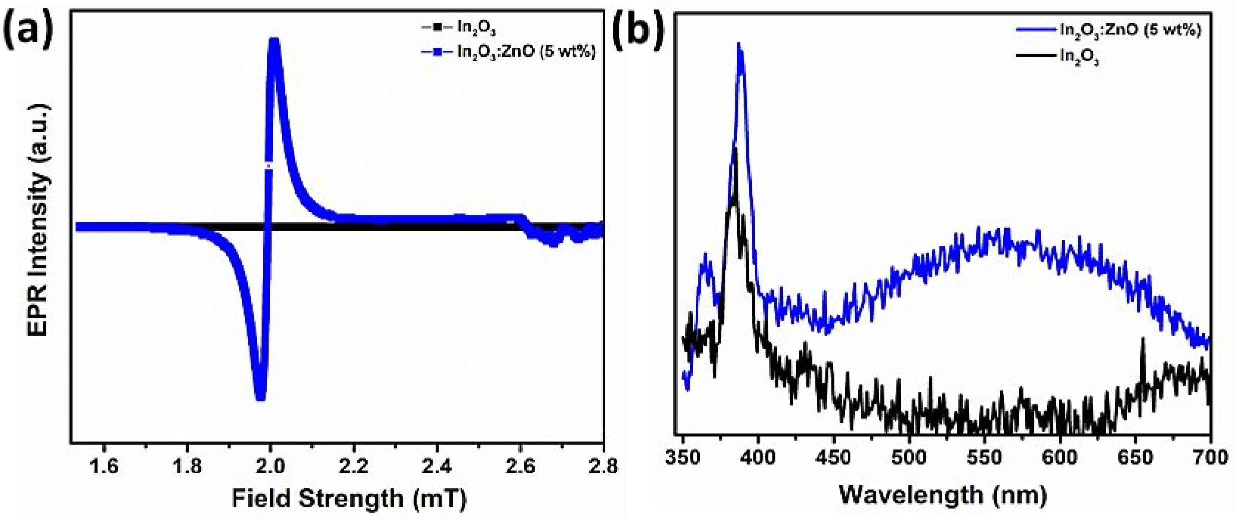

Figure S2. (a) EPR and (b) photoluminescence spectra measured at room temperature for In_2O_3:ZnO (5wt%) sample compared with only the In_2O_3 sample.

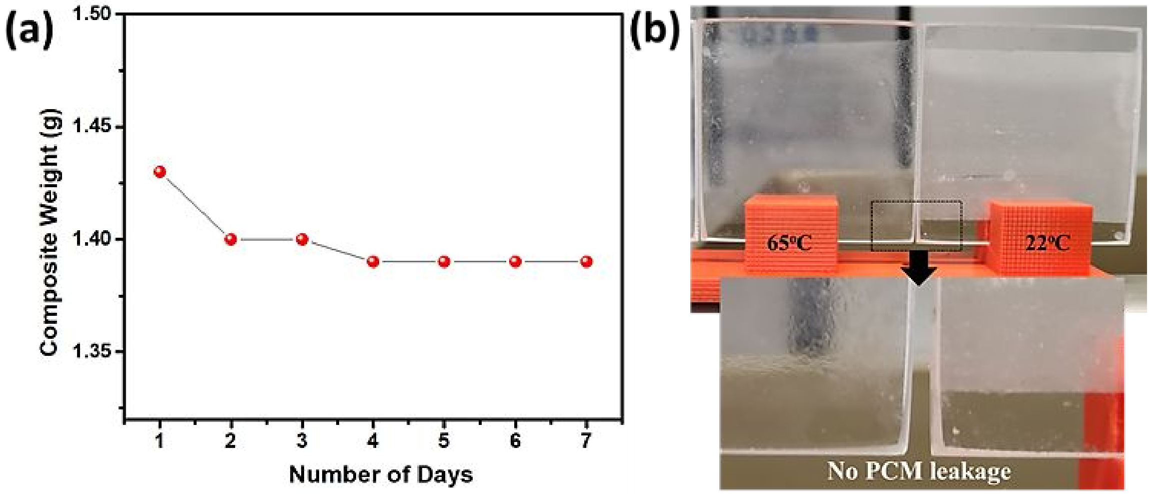

Figure S3. (a) Weight loss plot of the IZPC-5 composite coated film recorded for 7 days, indicating no weight loss of the coating when paraffin melts, and (b) corresponding photograph at different temperatures.
